# Prevalence and risk factors for falls in urban and rural older adults in Ekiti State, Nigeria

**DOI:** 10.4314/gmj.v55i4.6

**Published:** 2021-12

**Authors:** Oladele A Atoyebi, Olusegun Elegbede, Oluwole A Babatunde, Kayode Adewoye, Kabir Durowade, Dauda B Parakoyi

**Affiliations:** 1 Department of Community Medicine, Federal Teaching Hospital, Ido-Ekiti, Nigeria; 2 Department of Community Medicine, Afe Babalola University, Ado Ekiti, Nigeria; 3 Medical University of South Carolina, Charleston, USA

**Keywords:** older adults, falls, physical activity, ageing

## Abstract

**Objectives:**

This study assessed and compared the risk factors for falls among older adults in rural and urban communities.

**Design:**

A comparative cross-sectional approach was used.

**Setting:**

The study was conducted among community-living older adults in the rural and urban communities of the most populated Local Government Area (Ado-Ekiti LGA) in Ekiti State.

**Participants:**

The study population consisted of 624 persons aged 65 years and above recruited into rural and urban groups using multi-stage random sampling.

**Main outcome measures:**

Data collected using validated tools and physical measurements were subjected to binary logistic regression to determine the odds of falls with relevant predictor variables among older adults in both groups.

**Results:**

A significantly higher proportion of participants in the urban than the rural group had experienced a fall , and the associated risks include low visual acuity, increasing age, arthritis, hearing impairment, hyperglycaemia and high BMI. Physical activity was a protective factor.

**Conclusion:**

This study revealed a high risk of falls among older adults in the urban community. Early diagnosis and management of chronic conditions that increase fall risk and promote physical activity, especially among urban-dwelling older adults, are vital measures to be considered in fall prevention programmes.

**Funding:**

Self-funded research

## Introduction

Falling is a common serious condition that affects the health of older adults, and it is one of the most common geriatric syndromes threatening the independence of older persons.^1^ Although the actual definition of a fall in older people is open to some debate^2^, a frequently used definition is “unintentionally coming to the ground or some lower level and other than as a consequence of sustaining violent blow, loss of consciousness, sudden onset of paralysis as in stroke or an epileptic seizure.^3^ It has been estimated that one in three adults aged 65 and older falls each year, and 20% to 30% of those who fall suffer moderate to severe injuries, making it hard for them to get around or live independently, increasing their risk of early death.^4^,^5^ According to WHO, approximately 28–35% of people over the age of 65 fall each year, and this proportion increases to 32–42% for those aged more than 70 years.^6^

Falling among older adults results from a complex and interactive mix of biological or medical, behavioural and environmental factors, many of which are amenable to intervention. Research has identified many risk factors that contribute to falling, which are modifiable. Modifiable risk factors for falling identified in previous research include lower body weakness, difficulties with gait and balance, use of psychoactive medications, postural dizziness, poor vision, problems with feet and shoes, home hazards, lack of stair handrails, previous falls, poor stair design, muscle weakness, lack of bathroom grab bars, gait and balance problems, dim lighting or glare, poor vision, obstacles and tripping hazards, postural hypotension and slippery or uneven surfaces Other factors contributing to falls among older adults include chronic conditions including arthritis, diabetes, stroke, Parkinson's disease, incontinence, dementia, fear of falling, improper use of the assistive device and confusion.8 Research has shown that modifying these risk factors can significantly reduce rates of falling among older adult

Falls are the most frequent cause of bed-ridden conditions, injury-related morbidity, and mortality among older adults7 and are the leading cause of injury-related deaths in the ageing population. Falls cause considerable morbidity and mortality and affect the quality of life of many older adults. It is estimated that 1 out of 5 falls causes a serious injury such as head trauma or fracture, while every 29 minutes, an older adult dies from a fall. Many fall victims are often seriously injured, some are disabled, and falls may lead to post-fall syndrome, including increased dependence, loss of autonomy, confusion, immobilisation and depression. Within the year following a hip fracture from a fall, 20% of older people will die. A study conducted in Nigeria by Adebiyi et al. reported a fall rate of 21.4% among older adults in the year preceding Similarly, Olawale and Owoaje also reported that 25% of older persons in their study had various injuries from falls, which tied with traffic injuries as the leading cau Fall rates are associated with advancing age and disability.^4^ The Ibadan Study of Ageing reported a 10% disability among those aged 65–69 years.^16^

Furthermore, older persons who experience falls have increased health care utilisation, especially in low-resource settings. Falls may lead to further falls, and a history of previous falls is associated with fear of falling, leading to a restriction of activity, resulting in debility and elevated risk of future falls.^5^ Falling is common even among healthy older adults. For example, as many as 18% of well-functioning older men have fallen over one year.^17^,^18^ Even if a fall does not result in an injury, subsequent fear of falling may result in restricted activity. Perhaps the most damaging effect of falls is the physical and psychological impact of the fear of falling. Many older adults restrict their activities unnecessarily due to a fear of falls, which leads to a vicious cycle. Lack of physical activity causes a decrease in muscle tone and strength, leading to an increased risk of falling.^19^ Fall-related injuries are very common among older people and are a major cause of long-term pain and functional impairment. They also increase the risk of discharge to a nursing home and high economic costs.^20^

The direct costs that the older patients pay for treating fall-related injuries include fees for hospital and nursing home care, doctors and other professional services, surgery, rehabilitation, community-based services, use of medical equipment and prescription drugs while they incur indirect costs such as disability, dependence on others, lost time from work and household duties, and reduced quality of life.^21^ An analysis of research on preventing falls in older people showed that falls place a financial burden on the individual and the community.^22^ In 2010, falls among older adults cost the U.S. health care system $30 billion in direct medical costs; when adjusted for inflation, it was estimated that falls in those above 65 cost the NHS £4,600,000 each day.^23^ Fall injuries prolong hospitalisation and could cost up to $14,000 to manage.^24^

In Nigeria, an increasingly elderly population is at risk of falling, sustaining fall-related injuries and incurring the costs of treating these injuries. Few data exist on physical activity among older persons living in the community in Nigeria and its association with falls.

Injuries, particularly fall, among older adults are often unrecorded but are frequent events that may start a downward spiral in health status, resulting in death or longterm care needs.^6^ Older adults living in urban communities tend to have a different lifestyle than their counterparts in rural communities partly due to environmental factors, occupation trends and access to recreational facilities. Thus, the risk factors for falls are likely different in urban versus rural communities, and it is important to explore these disparities to target health promotion campaigns and policies accurately. Despite the increasing number of older persons and their attendant health problems on public health programs in developing countries, there is limited information on older adults about the main causes of falls, especially those residing outside major cities in Nigeria.^6^,^25^ This information would help initiate prevention strategies for falls had it been sufficient. In addition, the cost of preventable falls on the health care system would be better avoided if the falls were prevented. A preliminary search of the literature revealed that no study had been done to assess how the risk factors of falling among older persons in urban and rural settings vary in Nigeria. This research compares the risk factors for falls among older adults in rural and urban communities.

## Methods

This comparative cross-sectional study was carried out in Ekiti State, Nigeria. The study population consisted of persons aged 65 years and above living in the rural and urban communities of the most populated Local Government Area (Ado-Ekiti LGA). Consent was obtained from community-living seniors who were able to participate. A multi-stage sampling technique was used to select 624 respondents in this study with equal representation from rural and urban communities. Ado-Ekiti LGA is divided into 13 wards; 12 urban wards and one rural ward.^26^ In Stage 1 of the sampling, one of the twelve urban wards was selected using a simple random sampling technique by balloting.

This selected urban ward was compared with the only rural ward in the LGA. In stage 2, eight communities were selected from each urban and rural wards by balloting without replacement. In stage 3, proportionate allocation of calculated sample size was used to apportion the number of respondents to be sampled in each community selected in stage two. House numbering was done in each community. Using systematic random sampling, the first house was chosen randomly, and subsequent houses to be involved in the study were selected based on the sampling ratio. In the final stage, total sampling was used to recruit all those who met the study criteria in each house selected in stage 3 until the sample size was attained. Participants were invited to attend an information session at the community centre (rural group) and local hospital (urban group), where data were subsequently collected, and measurements taken. Data were collected using interviewer-administered questionnaires based on several standard and validated instruments.

The validated fall-risk self-assessment tool for older adults was used to assess the risk factors for falls.^27^ The tool has 12 questions with ‘yes’ and ‘no’ options. A ‘yes’ answer scores 1 point while a ‘no’ scores 0. The number of points was added up for each respondent, and those with a score of 4 points or more are at high risk for falling as defined in the fall risk assessment tool. Experience of falls was taken as falls occurring in the six months preceding the questionnaire administration.^28^ The WHO STEPwise approach to risk factor Surveillance (STEPS) instrument 29 was used to assess comorbidities and other risk factors for falls.

The Global Physical Activity Questionnaire 30 and Lawton's Instrumental Activities of Daily Living Scale^31^,^32^ were used to assess physical activity. For BMI calculations, the height of each respondent was measured with a mobile stadiometer, and the weight was measured using an Omron digital scale. Blood sugar, blood pressure (sitting and standing), and visual acuity measurements were taken. The blood pressure measurements (in the sitting and standing positions) were taken using the Omron M6 (HEM- 7001- E) for the left upper arm, which has been standardised for taking blood pressure measurements for the elderly at home.^33^,^34^ Fasting blood sugar was measured at 8 am with the one-touch ultra-glucometer, which has been standardised for taking blood sugar measurement at home, and visual acuity was measured with a Snellen's chart.

The Chi-square test was used to assess the association of categorical variables with falls experience between groups and determine the statistical significance of observed differences in cross-tabulated variables. Yates correction was applied where expected frequencies were small (<5) in any cells. Binary logistic regression was conducted to predict the risk of falling with relevant predictor variables for the respondents generally and rural and urban groups. Predictors were selected using the forced entry (enter) method, and the level of significance was predetermined at a p-value of less than 0.05 at 95% confidence level. Ethical approval for the study was obtained from the Ethical Review Committee of Federal Teaching Hospital, Ido-Ekiti, Nigeria (reference number ERC/2014/04/15/19A).

## Results

About a quarter of respondents (25.3%) in the rural group reported they had experienced a fall during the six months preceding this study. Conversely, a significantly higher percentage of participants (41.3%) in the urban group had experienced a fall (χ^2^=18.029, p<0.001). In the rural group, three-quarters of those aged ≥85 years reported falling during the preceding six months (Table 1). The prevalence of falls increased with age in the rural group, and different age groups were significantly different in terms of their falls experience (p<0.001). In the urban group, a similar upward trend in falls prevalence was observed with increasing age except that those aged 75–79 years accounted for the most falls in this group.

**Table 1:** Sociodemographic factors and fall experience of rural and urban respondents

Variable	Category	Rural		Urban		Inter-group analysis (participants who experienced a fall)
		Experienced a fall		Experienced a fall		
		No (%) n=233	Yes (%) n=79	No (%) n=183	Yes (%) n=129	
**Age group** **(Years)**	65–69	139 (90.3)	15 (9.7)	110 (69.2)	49 (30.8)	**χ^2^=22.104; p<0.001**
	70–74	41(77.4)	12 (22.6)	31(54.4)	26 (45.6)	
	75–79	33 (66.0)	17 (34.0)	18 (35.3)	33 (64.7)	
	80–84	13 (46.4)	15 (53.6)	14 (53.8)	12 (46.2)	
	≥85	7 (25.9)	20 (74.1)	10 (52.6)	9 (47.4)	
		χ^2^=67.720	p<0.001	χ^2^=19.703	p=0.001	
**Gender**	Male	130 (75.1)	43 (24.9)	81(66.4)	41 (33.6)	**χ^2^=10.438; p=0.001**
	Female	103 (74.1)	36 (25.9)	102 (53.7)	88 (46.3)	
		χ^2^=0.044	p=0.833	χ^2^=4.948	p=0.026	
**Marital Status**	Married	118 (88.1)	16 (11.9)	79 (65.8)	41 (34.2)	**χ^2^=5.401; p=0.020**
	Not Married	115 (60.85)	74 (39.15)	104 (54.17)	88 (45.83)	
		χ^2^=28.889	P<0.001	χ^2^=4.145	p=0.042	
**Highest level of education**	No Formal Education	151 (77.8)	43 (22.2)	36 (29.3)	87 (70.7)	**χ^2^=4.793; p=0.188**
	Primary	71 (71.7)	28 (28.3)	120 (79.0)	32 (21.0)	
	Secondary	9 (60.0)	6 (40.0)	11 (68.8)	5 (31.2)	
	Tertiary	2 (50.00)	2 (50.0)	16 (76.2)	5 (23.8)	
		χ^2^=4.479	p=0.214	χ^2^=72.945	p<0.001	
**Occupation in the last** **12months**	Trader	55 (65.5)	29 (34.5)	90 (52.0)	83 (48.0)	**χ^2^=22.719; p<0.001**
	Farmer	145 (81.5)	33 (18.5)	31 (63.3)	18 (36.7)	
	Artisan/ Technician	5 (50.0)	5 (50.0)	20 (71.4)	8 (28.6)	
	Civil Servant	4 (80.0)	1 (20.0)	5 (62.5)	3 (37.5)	
	Retired	24 (68.6)	11 (31.4)	37 (68.5)	17 (31.5)	
		χ^2^=12.078	p=0.017	χ^2^=7.666	p=0.105	

More female than male participants in both the rural and urban groups had experienced a fall. However, this gender disparity is higher and only significant in the urban group. In addition, the proportion of unmarried persons in both rural and urban groups who experienced a fall was higher than those that were married. These differences were more pronounced and significant in the rural group.

In the urban group, falls prevalence significantly decreased with increasing level of education (p<0.001) but this pattern is reversed in the rural group as the falls prevalence seemed to increase with increasing level of education. However, the observed difference in the level of education of those that had a fall was not statistically significant.

In the rural group, artisans/technicians were the working category with the most falls (50.0%) unlike in the urban group where traders sustained the most falls. Civil servants and artisans had the least proportion of older adults who fell in the rural group and urban groups respectively.

Table 2 shows the assessment of fall risk profile of respondents. Significantly, more urban and rural participants sometimes felt unsteady when walking, steadied themselves by holding on to furniture when walking at home, worried about falling, and needed to push with hands to stand up from a chair.

**Table 2:** Risk of fall profile in the rural and urban groups (n=312)

Fall risk factor	Category	Rural Frequency (%)	Urban Frequency (%)	χ^2^	p-value
**Use/advised using a cane or walker to get around safely**	No	285(91.3)	291(93.3)	0.813	0.367
	Yes	27 (8.7)	21(6.7)		
**Sometimes feel unsteady when walking**	No	277(88.8)	217(69.6)	34.980	<0.001
	Yes	35 (11.2)	95(30.4)		
**Steadies self by holding on to furniture when walking at** **home**	No	301(96.5)	251(80.4)	43.424	<0.001
	Yes	11(3.5)	61(19.6)		
**Worried about falling**	No	300(96.2)	255(81.7)	32.997	<0.001
	Yes	12(3.8)	57(18.3)		
**Need to push with hands to stand up from the chair**	No	279(89.4)	225(72.1)	30.086	<0.001
	Yes	33(10.6)	87(27.9)		
**Have some trouble stepping up onto a curb edge of a raised** **pavement/ sidewalk**	No	280(86.2)	224(71.8)	41.241	<0.001
	Yes	32(10.3)	88(28.2)		
**Lost some feeling in feet**	No	307(98.4)	259(83.0)	43.795	<0.001
	Yes	5(1.6)	53(17.0)		
**Takes medicine that sometimes makes feel light-headed or** **more tired than usual**	No	290(92.9)	287(92.0)	0.207	0.649
	Yes	22(7.1)	25(8.0)		
**Takes medicine to help sleep or improve mood**	No	268 (85.9)	235(75.3)	11.165	0.001
	Yes	44(14.1)	77(24.7)		
**Often feel sad or depressed**	No	306 (98.1)	263 (84.3)	36.868	<0.001
	Yes	6 (1.9)	49 (15.7)		

Also, more participants in the urban than the rural group had lost some feeling in their feet and had trouble stepping upon a curb edge of a raised pavement or sidewalk. Significantly more participants in the rural group took medicine to help them sleep or improve mood and were often feel sad or depressed.

Table 3 shows the odds ratio of the predictors of falls for respondents in the rural and urban groups. A test of the full model against a constant only model was statistically significant, indicating that the predictors as a set reliably distinguished between fallers and non-fallers (p < 0.001). In the rural group, the odds of falling were significantly increased with a high falls risk profile score (p=0.017), arthritis (p=0.001), and alcohol consumption (p=0.038), while the odds of falling significantly reduced with increasing Lawton's IADL score (p=0.017). However, in the urban group, the odds of falling were significantly increased with low visual acuity (p=0.014), increasing age (p=0.018), female gender (p=0.047), arthritis (p=0.044), hearing impairment (p=0.003), fasting blood sugar (p=0.002) and BMI (p=0.023) while the odds of falling were significantly reduced with increasing MET-minutes/week (p=0.038), Lawton's IADL score (p=0.021) and time for tandem stance (p<0.001).

**Table 3:** Factors contributing to falls among rural and urban respondents

	Rural			Urban		
Variables	Odds ratio	95.0% C.I.	p-value	Odds ratio	95.0% C.I.	p-value
**MET- minutes/week**						
**Low**	1	(reference)		1	(reference)	
**Adequate**	0.916	0.975 – 1.515	0.198	0.831	0.012 – 0.930	0.038
**IADL score**	0.816	0.083 – 1.792	0.049	0.578	0.231 – 0.907	0.021
**Falls risk score**						
**Low**	1	(reference)		1	(reference)	
**High**	17.226	5.889 – 50.791	0.017	1.326	0.854 – 2.059	0.208
**Tandem stance**						
**<10s**	1	(reference)		1	(reference)	
**≥10s**	0.020	0.000– 6.936	0.289	0.008	0.005 – 0.072	<0.001
**Visual acuity**						
**Normal**	1	(reference)		1	(reference)	
**Low**	3.347	0.090 – 12.393	0.512	1.062	1.041 – 1.129	0.014
**Blood pressure**						
**Normal**	1	(reference)		1	(reference)	
**High**	1.000	0.000 – 1.371	0.080	1.918	0.020 – 41.231	0.965
**Age**	17.453	0.789 – 38.587	0.057	1.013	1.009 – 1.477	0.018
**Gender**						
**Male**	1	(reference)		1	(reference)	
**Female**	1.003	0.037 – 2.615	0.093	1.601	1.308 – 8.218	0.047
**Monthly expenditure**						
**Below poverty line**	1	(reference)		1	(reference)	
**Above poverty line**	0.002	0.000 – 8.654	0.149	0.359	0.036 – 3.545	0.380
**Arthritis**						
**No**	1	(reference)		1	(reference)	
**Yes**	3.205	1.497 – 6.023	0.001	1.047	1.002 – 1.924	0.044
**Hearing impairment**						
**No**	1	(reference)		1	(reference)	
**Yes**	2.289	0.663 – 14.989	0.098	1.015	1.009 – 1.125	0.003
**Smoking cigarette**						
**No**	1	(reference)		1	(reference)	
**Yes**	2.289	0.663 – 14.989	0.098	0.003	0.000 – 0.000	0.997
**Alcohol consumption**						
**No**	1	(reference)		1	(reference)	
**Yes**	1.669	1.437 – 1.951	0.038	1.601	0.308 – 8.318	0.576
**Fasting blood sugar**						
**Normal**	1	(reference)		1	(reference)	
**High**	1.063	1.003 – 1.065	0.065	1.086	1.018 – 1.405	0.002
**BMI**						
**Normal**	1	(reference)		1	(reference)	
**Overweight**	1.664	0.081 – 1.703	0.703	1.105	1.016 – 1.734	0.023

## Discussion

A higher proportion of respondents in the urban than the rural group had experienced a fall in the six months preceding this study. The prevalence in the rural group is similar to what was reported in studies done previously in Ibadan, Nigeria (21.4%)^14^ as well as in other countries; Uruguay (27%), Argentina (28.5%), and Chile (34%).^35^

However, the prevalence of falls in the urban group in our study is higher than reported in previous research (27.6% in Brazil 5 and 37.4% in Ecuador).^18^ This disparity might be due to the difference in how falling was assessed or other salient differences in the study populations.

The prevalence of falls among respondents significantly increased with advancing age except in the urban group, where there is a spike among those aged 75–79 years. This increase in fall prevalence with age is statistically significant and conforms with findings from previous research^7^,^13^,^18^ According to a WHO report, 28–35% of people over the age of 65 fall each year, with this proportion increasing to 32–42% for those aged more than 70 years.^6^ The loss of muscle mass, coordination and frailty likely contribute to high falls with ageing. As humans age, the bodies experience many changes like poor vision, difficulties with gait, reducing muscle strength and endurance, and slower reaction time. These changes can lead to inactivity, which precludes loss of muscle strength, flexibility, coordination, balance, and posture, increasing a person's risk of falling.^4^

More females than males had experienced a fall in both study groups. A possible explanation is that men have bulkier muscle mass to help prevent falls by stabilising the joints.^36^ Generally, the risks of fall-related injuries are higher in women after 70 years.^18^,^36^ In both study groups, being without a spouse is significantly associated with falling, although the association was stronger in the rural group. This result might indicate that spouses are likely to have assistance with chores and duties that may otherwise make them less prone to falling.

The findings show that the falls are significantly associated with are no education, especially in the urban group. Formal education enhances awareness of risks, and the educated are likely to have adequate levels of physical activities, both of which deters falling.^37^ There is also an association between higher levels of education, socioeconomic status, physical activity and low risks of falling.^38^ The association between occupation and falls in the current study was significant only in the rural group. There were more retired persons than farmers who experienced a fall in this group. This might be due to the higher level of physical activity that farming involves.

The urban group's comparatively lower physical activity levels are associated with being obese or overweight and with higher fasting glucose levels, as seen among the urban participants. A cross-sectional study of older adults in Ilorin, Nigeria, revealed that body mass index (BMI) values between 20 and 24.9 are associated with optimum physical function and the lowest risk of morbidity. The authors emphasised that obesity leads to functionalism limitation among older adults.^39^ As we found, other studies have shown that fall risk is closely related to activities of daily living (ADLs) capability and that difficulty in at least one activity of daily living double the risk of falling.^40^ The risk of falling has been recorded to be higher among older adults with any impairment in ADLs.

Furthermore, regular alcohol use has also been associated with an increased risk of falling among older adults. There was a 1.4-fold higher risk of falling among older adults who drink on average two or more days per week than those who did not.^18^ The significant association between alcohol intake and falls in the rural group of the current study might have been due to a higher leisure consumption of local alcoholic products in this group.

### Limitations of the study

In addition to the measures of physical activity, we have explored several intrinsic risk factors for falls. This study was limited in not including extrinsic or environmental factors as covariates in our regression model. Accounting for environmental factors that affect rural vs urban dwellers would improve the reliability of the findings. In addition, polypharmacy or certain medications that predispose the user to falls could have to confound our results.

## Conclusion

Our findings show that more respondents in the rural than urban group had experienced a fall. Factors associated with falling include a high falls risk profile score, increasing age, female gender, and chronic conditions such as arthritis, low visual acuity, hearing impairment, high fasting blood sugar, and high BMI. Overall, the rural group had higher levels of protective factors against falls such as higher levels of physical activity and lower prevalence of obesity in comparison to the urban group.
